# Imbalance in the diurnal salivary testosterone/cortisol ratio in men with severe obstructive sleep apnea: an observational study^☆^

**DOI:** 10.1016/j.bjorl.2015.09.004

**Published:** 2015-12-17

**Authors:** Cristina Mihaela Ghiciuc, Lucia Corina Dima-Cozma, Raluca Mihaela Bercea, Catalina Elena Lupusoru, Traian Mihaescu, Sebastian Cozma, Francesca Romana Patacchioli

**Affiliations:** a University of Medicine and Pharmacy Grigore T. Popa, Department of Pharmacology, Iasi, Romania; b University of Medicine and Pharmacy Grigore T. Popa, School of Medicine, Department of Internal Medicine, Iasi, Romania; c University of Medicine and Pharmacy Grigore T. Popa, Clinic of Pulmonary Diseases, Iasi, Romania; d University of Medicine and Pharmacy Grigore T. Popa, Department of Otorhinolaryngology, Iasi, Romania; e Sapienza University of Rome, Department of Physiology and Pharmacology “V. Erspamer”, Rome, Italy

**Keywords:** Obstructive sleep apnea, Saliva, Cortisol, Testosterone, Apneia obstrutiva do sono, Saliva, Cortisol, Testosterona

## Abstract

**Introduction:**

The complex relationship between sleep disorders and hormones could lead to alterations in the production of cortisol and testosterone in obstructive sleep apnea (OSA) patients.

**Objective:**

The purpose of this study was to determine the diurnal trajectories of salivary free-testosterone, free-cortisol and their ratio (T/C).

**Methods:**

Ten subjects newly diagnosed with OSA, based on nocturnal polysomnography evaluation and excessive daytime sleepiness, and seven matched controls were consecutively recruited. Cortisol and testosterone were measured in salivary samples collected upon awakening, at noon and in the evening. The psychometric evaluation of anxiety/depression and referred sexual function disturbances was performed to evaluate the presence of neuropsychological comorbidities.

**Results and conclusion:**

The main finding was that OSA subjects displayed hypocortisolism upon awakening and a significant reduction in testosterone concentration in the evening in comparison with the control group, which has maintained the physiological testosterone and cortisol diurnal fluctuation, with higher hormone concentrations in the morning and lower concentrations in the evening. The use of data from multiple diurnal measurements rather than a single point allowed the detection of T/C ratio changes of opposite signs at the beginning and end of the day: the OSA subjects had a higher T/C ratio than the controls in the morning, while their T/C ratio was significantly lower than that of the controls in the evening. The imbalances in the anabolic-catabolic diurnal equilibrium suggest that OSA is associated with a dysregulation of the hypothalamic–pituitary–adrenal and hypothalamic–pituitary–gonadal axes, potentially an underlying cause of some of the neuropsychological comorbidities observed in OSA patients.

## Resumo

**Introdução:**

A relação complexa entre os distúrbios do sono e os hormônios pode levar a alterações na produção de cortisol e testosterona em pacientes com Apneia obstrutiva do sono (AOS).

**Objetivo:**

O objetivo deste estudo foi determinar as curvas diurnas de testosterona e cortisol livres na saliva e sua proporção (razão T/C).

**Método:**

Dez indivíduos recém-diagnosticados com AOS com base na avaliação por polissonografia noturna e sonolência diurna excessiva e sete controles pareados foram recrutados, consecutivamente. Cortisol e testosterona foram medidos em amostras de saliva coletadas ao acordar, ao meio-dia e à noite. A avaliação psicométrica dos distúrbios de ansiedade/depressão e função sexual mencionados foi realizada para detectar a presença de comorbidades neuropsicológicas.

**Resultados:**

O achado principal foi que os indivíduos com AOS apresentam hipocortisolismo ao acordar e uma redução significante na concentração de testosterona à noite, em comparação com o grupo controle, que manteve a variação fisiológica diurna de testosterona e cortisol com concentrações hormonais mais elevadas pela manhã e concentrações mais baixas durante a noite. O uso de dados de várias mensurações diurnas, em vez de uma única mensuração, permitiu detectar as alterações na razão T/C de sinais opostos no início e no final do dia: os indivíduos com AOS apresentaram razão T/C maior que os controles na parte da manhã, enquanto que a razão T/C foi significantemente inferior à dos controles durante a noite.

**Conclusão:**

Os desequilíbrios no balanço anabólico-catabólico diurno sugerem que a AOS está associada a uma desregulação dos eixos hipotálamo-hipófise-adrenal e hipotálamo-hipófise-gonadal, potencialmente a causa subjacente de algumas das comorbidades neuropsicológicas observadas em pacientes com AOS.

## Introduction

Obstructive sleep apnea (OSA) is a chronic respiratory disturbance that can be diagnosed with an overnight polysomnography (PSG). It is a serious health problem with prevalence greater than 26% in the general middle-aged population.^1^, ^2^, ^3^

Excessive daytime sleepiness and snoring are often associated with neuropsychological symptoms, including concentration difficulties and cognitive disturbances. Hypoxia, sleep fragmentation, obesity, and ageing in OSA patients were found to be associated with low serum testosterone levels. Luboshitzky et al.^4^ demonstrated that men with sleep apnea had decreased levels of testosterone and luteinizing hormone during nightly sleep, which is possibly caused by the combined effects of hypoxia and sleep fragmentation. More recently, Bercea and co-workers reported that the morning serum testosterone concentration in obese patients with severe OSA was associated with increased anxiety/depression and sleep efficiency disturbances.^5^

The complex relationship between nocturnal sleep disorders and the hypothalamic–pituitary–adrenal (HPA) axis could lead to alterations in the production of cortisol.^6^, ^7^, ^8^, ^9^ Testosterone and cortisol, major end products of the hypothalamic–pituitary–gonadal (HPG) axis and the HPA axis, respectively, are part of a biological balance that modulates psychologically and physically integrated human responses. The interrelationship between sex and stress hormones has been extensively investigated in the context of sports medicine by measuring the testosterone/cortisol (T/C) ratio as a marker of anabolic/catabolic activities connected with athlete performance and overtraining.^10^, ^11^ Furthermore, the T/C ratio has been proposed as a hormonal marker of psychopathologies,^12^, ^13^ and a specific association between a reduction of the T/C ratio and ischemic disease has also been reported.^14^

The purpose of this study was to determine the diurnal fluctuation of salivary free-testosterone (T) and free-cortisol (C) and to study their interrelationship by measuring diurnal variations in the T/C ratio of patients with severe OSA. This analysis will expand upon previous studies that provided only partial data from single-point hormonal measurements.

Interestingly, sex hormone imbalance is often associated with depressive and anxious mood disorders in men^15^ and bidirectionally interacts with erectile function.^16^ Therefore, to assess the presence of comorbidities, the Hamilton Inventory Questionnaires for Depression (HDS) and Anxiety (HAS) and the International Index for Erectile Function (IIEF) questionnaire were administered to the study population.

## Materials and methods

### Study population

This prospective study was conducted over 6 months between May 2011 and December 2012 in the Sleep Laboratory in the Clinic of Pulmonary Diseases (Iasi, Romania). The study was formally approved by the local Ethics Committee (Protocol n° 14, April 29th, 2011). All Caucasian subjects were recruited among patients visiting the Centre for Sleep Disturbances and provided their written informed consent before the start of the study.

We previously estimated that at least 14 subjects (7 per group) were required to detect a mean absolute difference corresponding to a 50% variation on the expected peak of T/C ratio in healthy subjects (40.00 ± 20.00 arbitrary unit) with a two-tailed *α* of 0.05 and 80% of power analyses. Thus, 10 patients (OSA group) were selected based on the following inclusion criteria: male, 40–60 years old, non-smoker, with body mass index (BMI) >30 kg/m^2^ (obese) and newly diagnosed with severe OSA based on nocturnal polysomnography (PSG) evaluation (apnea-hypoapnea index, AHI ≥ 30 h^−1^) and excessive daytime sleepiness (Epworth Sleepiness Scale, ESS ≥ 10).^17^ The exclusion criteria included the following: acute or chronic associated diseases, smoking, use of any chronic medication and non-cooperative attitude. Patients with suspected OSA were hospitalized for 4 consecutive days. On the morning of admittance, blood samples were collected for biochemical-hematological parameter determinations, and pulmonary function assessment (spirometry) and psychometric evaluations with the Hamilton Rating Scale for Depression,^18^ with the Hamilton Anxiety Rating Scale and with the International Index Erectile Function questionnaire (IIEF)^19^ were performed. A baseline resting electrocardiogram (ECG), heart rate (HR) (BTL-08 USA) and systolic and diastolic blood pressure were recorded (M_3_ – Omron, Japan). Conventional sleep recordings (SOMNOlab V2.01, Weinmann, Germany) were obtained from 10:00 pm to 7:00 am (day 2) and AHI, arterial oxygen saturation (SpO_2_), desaturation index (ODI) and microarousal index, were scored applying standard criteria.^20^ According to American Academy of Sleep Medicine 2007, the AHI was calculated as the mean number of apneas plus hypopneas per hour of sleep; apneas were scored when the absence of airflow was lasting at least 10 s and hypopneas when there was at least a 30% drop in the oronasal airflow, with coincident oxygen desaturation of at least 4%, lasting at least 10 s.^7^, ^20^

The participants were instructed on how to collect saliva and asked to avoid food, coffee and alcohol consumption, teeth brushing and any physical exercise for at least 30 min before each saliva collection.^21^ Thus, on the day after (day 3), saliva was collected upon awakening (between 6:30 h and 7:30 h), at noon (before lunch) and in the evening at 19:00 h before dinner, for measuring the daily cortisol and testosterone fluctuation. The exact time of saliva sampling was monitored by a staff member. Among the patients hospitalized with suspected OSA, we selected 7 adult male subjects for the control group who had the same somatic characteristics of the OSA subjects but did not suffer from sleep apnea (AHI < 5 h^−1^, ESS < 10).

### Salivary sampling procedure, testosterone and cortisol assay

Saliva was collected using the Salivette (Sarstedt, Italy) sampling device, which allows for quick and hygienic saliva recovery through centrifugation at 3000 rpm for 15 min.^22^

For each sample, duplicate measurements were performed for the direct assay of testosterone on 100 μL of saliva (inter-assay coefficient of variation was <10%, and intra-assay coefficient of variation <7% with a minimum detectable concentration of 3.5 pg/mL at the 95% confidence limit using commercial immunoenzymatic kits and for the direct assay of cortisol on 25 μL of saliva (inter-assay coefficient of variation was <10%, and intra-assay coefficient of variation <7% with a minimum detectable concentration of 0.5 ng/mL at the 95% confidence limit (Diametra, Italy).

### Data analysis

All data were reported as the mean ± SEM. The statistical analyses were performed and the graphics were produced using the SigmaPlot 11 software package (SxST.it, Italy). Normal distribution of the data was tested using the Kolmogorov–Smirnov test. Where appropriate, Student's *t*-test or the Mann–Whitney *U* test was used as parametric and non-parametric tests, respectively, for the comparisons between groups.

A two-way ANOVA followed by the Fisher LSD method for post-hoc multiple-comparison tests were performed to reveal “GROUP”, “TIME”, and “GROUP × TIME” effects on T, C, and the T/C ratio measured in both groups at 7:00, 12:00, and 19:00 on the sampling day.

The area under the curve (AUC) was calculated from the AUC determined for each subject by the trapezoidal method using the three salivary testosterone values measured during the sampling day (morning, noon, and evening).^23^

Statistical significance was set at *p* *<* 0.05.

## Results

### Characteristics of the study population

As reported in Table 1, the two groups were matched for age, and there were no significant differences in the BMI values; the mean waist size was similar for both groups and was however above the normal range for men.

**Table 1:** Somatic, polysomnographic, and psychometric variables in the study population.

	Control (*n* = 7)	OSA (*n* = 10)	Statistics	*p*-Value
Age (years)	51 ± 3	53 ± 3	*T* = 58.000	0.66
BMI (kg/m^2^)	32.2 ± 0.6	32.3 ± 0.7	*T* = 69.000	0.591
Waist circumference (cm)	105 ± 2	109 ± 2	*t* = −1.623	0.125

*Polysomnography parameters*				
AHI (h^−1^)	2.57 ± 0.48	63.5 ± 9.3	*T* = 28.000	<0.001
Minimum SpO_2_ (%)	87.6 ± 1.49	68.5 ± 4.27	*T* = 95.000	0.002
ODI (h-1)	4.86 ± 0.83	58.4 ± 9.35	*T* = 28.000	<0.001
Sleep duration (min)	419.3 ± 8.5	395.3 ± 7.5	*t* = 2.088	0.054
Sleep efficiency (%)	79.9 ± 0.7	78.6 ± 0.7	*t* = 1.370	0.191
Microarousal (h-1)	9.9 ± 0.94	46.7 ± 6.31	*T* = 28.000	<0.001
ESS	4.0 ± 0.83	12.9 ± 1.23	*t* = −4.910	<0.001

*Systemic blood pressure and HR*				
SBP (mmHg)	117 ± 3	127 ± 2	*t* = −2.744	<0.05
DBP (mmHg)	69 ± 4	76 ± 2	*t* = −1.657	0.118
Heart rate (beats/min)	67 ± 1	75 ± 2	*T* = 48.000	0.156

*Psychometric scores*				
HDS	5 ± 1	10 ± 0.54	*t* = −5.073	<0.001
HAS	2 ± 1	5.3 ± 1	*T* = 38.000	<0.05
EF	27.9 ± 0.7	16.0 ± 0.9	*T* = 98.000	<0.001
OF	9.0 ± 0.2	6.9 ± 0.3	*T* = 97.000	<0.001
SD	9.1 ± 0.1	6.9 ± 0.2	*T* = 98.000	<0.001
IS	14.0 ± 0.2	6.5 ± 0.2	*t* = 18.190	<0.001
OS	10.0 ± 0	8.5 ± 0.2	*T* = 98.000	<0.001

Data are expressed as the mean ± SEM. BMI, body mass index; AHI, apnea-hypopnea index; minimum SpO_2_, minimum oxygen saturation; ODI, oxygen desaturation index; ESS, Epworth Sleepiness Scale; SBP, systolic blood pressure; DBP, diastolic blood pressure; HDS, Hamilton Depression Score; HAS, Hamilton Anxiety Score; EF, erectile function, OF, orgasmic function; SD, sexual desire; IS, intercourse satisfaction; OS, overall satisfaction.

The results of the pulmonary function tests (spirometry), forced expiratory volume (FEV), and vital capacity (FEV/VC ratio) were within the normal limits for subjects in both groups (data not reported). As shown in Table 1, all PSG parameters were within normal ranges in the control group. In contrast, the OSA group had significantly higher AHI values and a significant decrease in the minimum SpO_2_, which is a characteristic of severe OSA. The ODI was also significantly increased in the OSA group. The shorter duration of sleep in OSA subjects does not reach statistical significance and no changes have been detected in the sleep efficency. Moreover, the number of microarousal events was significantly higher in the OSA group than in the control group. In addition, excessive daytime sleepiness, as evaluated by the ESS, was significantly higher in the OSA group than in the control group.

Table 1 also shows that the mean systolic blood pressure was slightly elevated in the OSA group compared to the control group, with no change in diastolic blood pressure. In addition, the OSA patients showed a tendency toward an increased HR.

The Hamilton Inventory Scores for Depression (HDS) and Anxiety (HAS) and the IIEF results for the study population are also reported in Table 1. OSA patients had significantly higher psychometric scores for anxiety and depression than the controls. The psychometric scores of the control group indicated no signs of depression or anxiety. Furthermore, the OSA patients were significantly more likely to have erectile function disorders than the controls.

Individual hematological and serum biochemical markers of glycemia and lipids (total cholesterol and triglycerides) were within the normal ranges for both groups (data not shown).

### Diurnal trajectories of salivary free-testosterone and salivary free-cortisol in the study population

**Figure 1:**
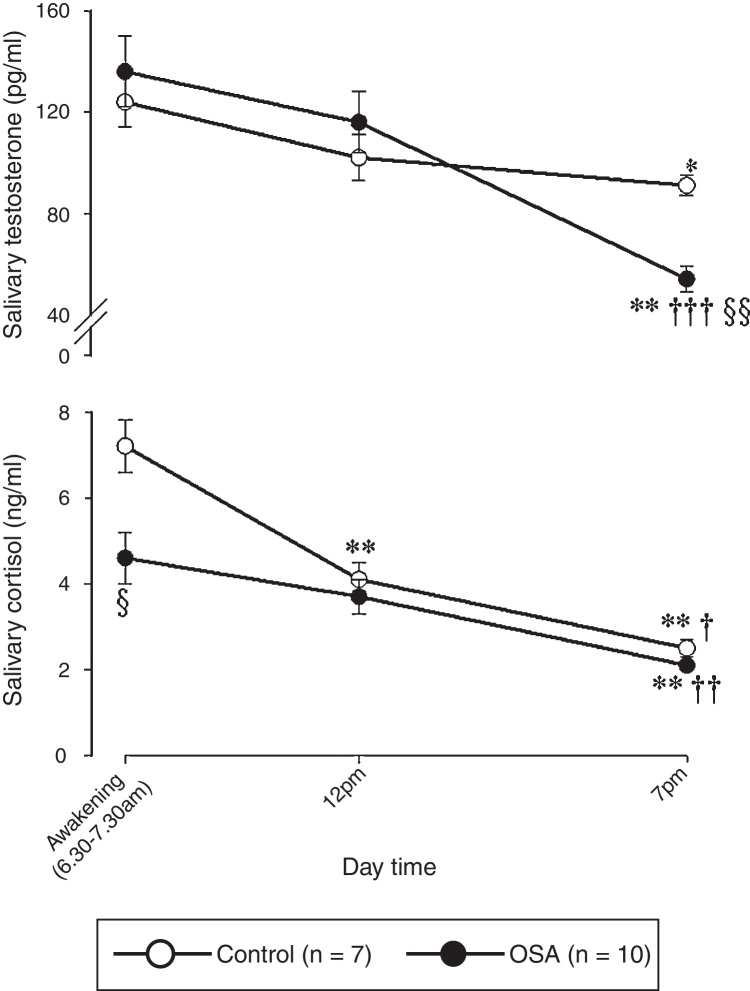
Diurnal trajectories of salivary free-testosterone and salivary free-cortisol in the study population. The data are presented as the mean ± SEM. Statistical analysis: a two-way ANOVA followed by post-hoc test for multiple comparisons: * and **: *p* < 0.05 and *p* < 0.01, respectively, vs. the value at 07:00; ^†^, ^††^, and ^†††^: *p* < 0.05, *p* < 0.01, and *p* < 0.001, respectively, vs. the value at 12:00; ^§^ and ^§§^: *p* < 0.05 and *p* < 0.01, respectively, vs. the control.

Fig. 1 shows the salivary free-testosterone (upper panel) and salivary free-cortisol (lower panel) daily trajectories for the study population.

A two-way ANOVA analysis showed that there were significant differences in the salivary testosterone concentrations measured at different times of the day (GROUP: *F*_1,50_ = 0.184, *p* = 0.670; TIME: *F*_2,50_ = 15.350, *p* *<* 0.001; GROUP × TIME interaction: *F*_2,50_ = 3.701, *p* = 0.032). The post-hoc test for multiple comparisons showed that the Testosterone concentration measured in the evening for the control group (91 ± 4 pg/mL) was significantly lower than the value in the morning (124 ± 10 pg/mL, *p* *<* 0.05). The same diurnal fluctuation was detected in the OSA group; the Testosterone in the evening (54 ± 5 pg/mL) was significantly lower than that in the morning (136 ± 14 pg/mL, *p* *<* 0.001). In addition, the Testosterone concentration in the OSA group at 19:00 was significantly lower (*p* *<* 0.01) than the Testosterone concentration in the control group. Finally, no significant difference was detected between the control group and the OSA group in the AUC computed from the diurnal total Testosterone production (1240 ± 53 pg/mL/h and 1222 ± 108 pg/mL/h, respectively).

The C concentrations in the control and OSA groups are reported in Fig. 1. A two-way ANOVA demonstrated significant differences between and within the subject groups (GROUP: *F*_1,50_ = 9.594, *p* = 0.003; TIME: *F*_2,50_ = 33.152, *p* *<* 0.001; GROUP × TIME interaction: *F*_2,50_ = 3.753, *p* = 0.031).

In both the control and OSA groups, the salivary Cortisol concentration in the morning (7.2 ± 0.6 ng/mL and 4.6 ± 1.0 ng/mL, respectively) was significantly higher than that in the evening (2.5 ± 0.4 ng/mL, *p* *<* 0.001 and 2.1 ± 0.1 ng/mL, *p* *<* 0.001, respectively). Furthermore, in the morning, the Cortisol concentration in the OSA group was significantly lower than that in the control group (*p* *<* 0.05).

### Diurnal trajectories of salivary T/C ratio in the study population

**Figure 2:**
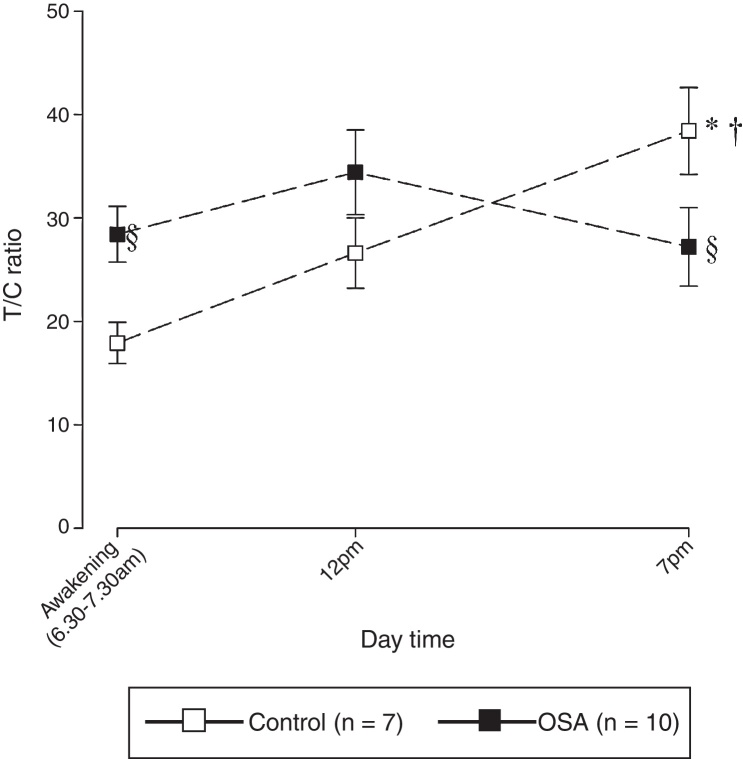
Diurnal trajectories of the T/C ratio in the study population. The data are presented as the mean ± SEM. Statistical analysis: a two-way ANOVA followed by post-hoc test for multiple comparisons, * *p* < 0.001 vs. the value at 7 am; ^†^*p* < 0.05 vs. the value at 12 pm; ^§^*p* < 0.05 vs. the corresponding control.

Fig. 2 depicts the diurnal trajectories of the T/C ratio in the study population. A two-way ANOVA demonstrated significant differences in the T/C ratio measured during the sampling day (GROUP: *F*_1,50_ = 0.633, *p* = 0.431; TIME: *F*_2,50_ = 3.880, *p* = 0.028; GROUP × TIME interaction: *F*_2,50_ = 5.347, *p* = 0.008). In the control group, there was a progressive and statistically significant increase in the T/C ratio during the sampling day (7:00 = 18 ± 9; 12:00 = 27 ± 3; 19:00 = 43 ± 5). In contrast, there were no significant diurnal fluctuations in the T/C ratio in the OSA group (7:00 = 28 ± 3; 12:00 = 34 ± 4; 19:00 = 27 ± 4). Additionally, the T/C ratio in the OSA group was significantly higher than that in the control group in the morning (*p* *<* 0.05) and significantly lower than that in the control group in the evening (*p* *<* 0.05).

## Discussion

In this study, we measured the diurnal trajectories of the salivary free-testosterone and Cortisol concentrations and analyzed the interaction of these two hormones by assessing the diurnal variation of the T/C ratio in patients with severe OSA. In addition, we performed a psychometric evaluation of anxiety/depression and self-reported sexual function disturbances. The major finding of this study is that male obese OSA patients have a reduced T concentration in the evening and a reduced C concentration in the morning compared with non-apneic obese controls. Furthermore, male obese OSA patients have an imbalance in the T/C ratio measured in the morning and in the evening.

Sex hormones are believed to be involved in the pathogenesis of OSA in adult male subjects who present with reduced levels of circulating androgens.^5^, ^16^ In the present study, there were no differences between the control and OSA groups in the fluctuation of the diurnal T trajectory; the evening T concentration was typically lower than the morning concentrations in both experimental groups.^24^, ^25^ Salivary testosterone concentration lower than controls also translates in an increase in comorbidities (fatigue, depression, general reduction of the well-being sense, sleep disturbances, etc.)^16^ affecting our OSA subjects. Since BMI above the normal range was present in both experimental groups, OSA itself, rather than obesity, seems to be the cause of the drop of salivary testosterone concentration in the present study. It has been suggested that the morning rise in testosterone production is derived from an endogenous testosterone circadian rhythm and is not directly related to any of the sleep stages.^26^ However, the OSA patients in this study experienced a decrease in the lead to stressful modifications of the sleep patterns with a decreased sleep efficiency.^26^

In this study, we demonstrated that OSA subjects maintain physiological circadian activity in the HPA axis, with the highest cortisol concentrations present in the morning and the lowest present in the evening. The maintenance of regular circadian cortisol production in OSA subjects has also been reported in a previous study^27^; however, these authors did not detect any difference between the OSA patients and controls in the morning cortisol concentration. This discordance may be related to the fact that in the present study is detected in the saliva only the “bioactive” free fraction, which corresponds to the unbound free plasma fraction of the hormone.^7^ Indeed, morning hypocortisolism in OSA patients has been recently confirmed.^8^ The highest T and C concentrations occur at the beginning of the day, while the lowest levels occur in the evening.^25^, ^28^ In our study, we used a three-point diurnal sampling schedule for the first time and were able to detect a gradual increase in the T/C ratio throughout the day. A change in the balance between the two steroid hormones may put an individual at risk for a breakdown in homeostasis. In the present study, the OSA subjects had a higher morning T/C ratio than the controls; however, in the evening, the T/C ratio was significantly lower in the OSA subjects compared with the control group. This imbalance in the anabolic-catabolic equilibrium could be attributed to the morning hypocortisolism that characterizes severe OSA^7^, ^8^; the resultant flattened response to chronically repeated nocturnal challenges reflects a dysregulation of the HPA axis.^29^ In contrast, the decrease in the T/C ratio in the OSA group in the evening could be a result of the sharp and marked drop in the T concentration at 19:00.

Obesity in men is associated with reduced androgen secretion.^30^ However, in the present study, obesity was not considered a confounding factor in the T concentration reduction because obesity occurred in both groups. Treatment of moderate to severe OSA with continuous positive airway pressure (CPAP) does not reliably increase testosterone levels in most studies. In contrast, a reduction in weight does so predictably and linearly in proportion to the amount of weight lost.^16^

Compared with the control group, the OSA patients demonstrated either a mild anxious-depressive disorder or excessive daytime sleepiness. Recently, a relationship between a depressive mood and obesity was described.^31^ However, the contribution of the T/C ratio imbalance to the development of anxious-depressive symptoms in patients with severe OSA and the key role that obesity plays in the pathogenesis of OSA comorbidities needs to be further studied.

OSA is a recognized cause of sexual disturbances in men.^16^, ^32^, ^33^ In this study, we confirmed that OSA patients suffer from mild to moderate sexual dysfunction as assessed by the IIEF questionnaire.^19^

## Conclusions

The main finding of this study is that male obese OSA patients have significant changes in the T and C diurnal trajectories in comparison with non-apneic obese controls. Hypocortisolism was particularly pronounced in the morning. Conversely, the T concentration was lowest in the evening. Moreover, although OSA patients maintained the physiological diurnal T/C ratio fluctuation, they exhibited an imbalance in the diurnal T/C ratio trajectory.

This study improved on other previous studies by using salivary hormone measurements to detect the free fraction of steroid hormones in the plasma^34^ and by employing a targeted protocol. In particular, the use of data from multiple diurnal measurements rather than a single point permitted the detection of T/C ratio changes of opposite signs at the beginning and end of the day, allowing for a more accurate study of the HPG and HPA axes. Finally, it is likely that in OSA subjects, poor sleep quality, tiredness, and psychological distress are among the comorbidities affecting the responses crucial to the maintenance of the physiological anabolic-catabolic hormonal balance. However, we did not establish a causal relationship between these factors. In fact, a limitation of this study is that we only demonstrated the co-existence of imbalanced T and C production and OSA comorbidities. To fully address this possible relationship, further research, with sample sizes adequate for a correlative study design, is necessary.

## Conflicts of interest

The authors declare no conflicts of interest.
